# Cleanliness

**Published:** 1892-04

**Authors:** 


### CLEANLINESS.

Habits of cleanliness have much to do with preserving good health. Thousands of persons are now on the sick list for want of cleanliness, and many are killed by its neglect. For convenience sake we will divide our subject into two parts—that treating of general cleanliness, and that of particular cleanliness. By general cleanliness I mean that of our bodies and surroundings. Every person should keep his skin clean by means of a daily dry rub and a weekly hot bath or wash all over with warm water. Besides this, he should change his underclothes daily; the shirt worn in the day should be removed at night and a night-dress worn ; this night-dress may be of flannel if desired. When a person rises from his bed in the morning he should remove the dress worn in the night, and throw it over the back of a chair, so that it may be thoroughly aired before it is again put on. Next, he should throw the bedclothes back over the rail at the foot of the bed, and so expose them to the pure air. Some are more particular, and throw each sheet or blanket over a separate chair, and thus make sure of perfect exposure of their bed linen to the air. All slops should be removed from a bedroom as soon as possible, so that the room may be kept sweet. Some persons go so far as to provide a cover for their chamber utensils. This is a step in the right direction, and prevents the air of a bedroom being unduly fouled.

Sponges, flannels, or other articles used in keeping the body clean should often be washed in boiling water, and exposed to the air to keep them sweet and clean.

In the living-room the windows should always be kept open a little in winter and more in summer, to ensure that the air we breathe is kept pure. We have no more right to breathe again the foul breath from our lungs than we have to eat the refuse food that has passed through our bodies. Foul air clogs up our lungs, renders our blood impure, and so shortens life. Wherever we are we must never forget that the air we expel from our lungs is very unclean and unfit for further re-breathing ; to breathe pure air night and day as much as possible through our noses must be our constant care if we would have clean and healthy lungs.

The clothes we wear should be kept clean by being brushed daily if possible. Those who are engaged in dirty or dusty work should have their clothes well beaten every day after they come home from work. This applies specially to those who are employed in chemical works. Those who work at greasy occupations should have the grease removed from their clothes by a little benzine. If these simple precautions are taken better health will result, as dust and disease germs will then be kept as much as possible outside the home.

In dusting a room be careful that the dust disturbed does not settle down again or cling to the wall. It is best to use a slightly moist duster for going over furniture, and then a dry one afterwards. By this means the dust is retained by the moist duster, and can be washed out of it afterwards. If a dry duster is used, then it should be shaken out of the window every few minutes. It is a good plan, if living in the country, to take every article out of doors and give it a good dusting first and an airing afterwards.

